# From *ex vivo* to *in vitro* models: towards a novel approach to investigate the efficacy of immunotherapies on exhausted Vγ9Vδ2 T cells?

**DOI:** 10.3389/fimmu.2025.1556982

**Published:** 2025-04-22

**Authors:** Morgane Chauvet, Dorothée Bourges, Emmanuel Scotet

**Affiliations:** ^1^ Nantes Université, Inserm UMR 1307, CNRS UMR 6075, Université d’AngersCRCI2NA, Nantes, France; ^2^ LabEx IGO “Immunotherapy, Graft, Oncology”, Nantes, France; ^3^ Sanofi, Oncology, Vitry-sur-Seine, France

**Keywords:** T cell exhaustion, cancer, Vγ9Vδ2 T cell, *in vitro* models, immunotherapy

## Abstract

Human γδ T cells demonstrate remarkable and diverse antitumor properties driven by TCR-dependent activation. Their non-alloreactive nature and pivotal role in cancer immunity position them as attractive targets for immunotherapies. However, upon infiltrating tumors, due to mechanisms induced by the tumor microenvironment’s immune evasion strategies, these cells frequently become exhausted, greatly weakening the efficacy and antitumor potential of novel immunotherapeutic treatments. While being extensively characterized in CD8^+^ T cells, research on γδ T cell exhaustion remains scarce. There is a growing need for comprehensive models to investigate the reinvigoration properties of exhausted γδ T cells. This review synthesizes current strategies and models for evaluating novel immunotherapies aimed at rejuvenating exhausted γδ T cells. It explores a progression of approaches, from *ex vivo* studies and *in vivo* murine models to emerging *in vitro* systems. The advantages and limitations of these models are discussed to provide a comprehensive understanding of their potential in advancing therapeutic research. Furthermore, recent findings suggesting *in vitro* exhaustion phenotypes closely mirror those observed *ex vivo* highlight opportunities for preclinical innovation. By refining these models, researchers can better optimize the immunotherapies targeting this unique T cell subset.

## Highlights

- *Ex vivo* Models
  These involve direct collection of exhausted T cells from patient samples, providing the closest representation of tumor microenvironment conditions. However, they are limited by the availability of samples, the variability of the tumors, a low cell yield, and the inherent heterogeneity of the cells.
- Murine Models
  Widely used due to their flexibility and established protocols, murine models enable the study of T cell exhaustion mechanisms without relying on human samples. However, the lack of Vγ9Vδ2 T cells or a human tumor microenvironment and key species-specific differences restrict their applicability for studying γδ T cells.
- Non-Human Primate Models
  These models offer the closest immune system approximation to humans, enabling *in vivo* study of Vγ9Vδ2 T cell exhaustion. Nonetheless, their lack of tumor models, high cost, complexity, and ethical considerations limit their routine use.
- Humanized Mouse Models
  Immunodeficient mice engrafted with human immune components allow partial reconstitution of the human immune system, including Vγ9Vδ2 T cells. While promising for translational research, challenges such as incomplete immune system representation, cytokine dependency for cell survival, and high cost remain significant.
- *In vitro* Models
  These models use expanded T cells exposed to conditions mimicking exhaustion, such as sustained stimulation, hypoxia, or nutrient deprivation. They are highly customizable and capable of producing large numbers of cells for high-throughput testing. However, they struggle to replicate the full complexity of the tumor microenvironment and lack sufficient data for Vγ9Vδ2-specific protocols.

## 1 Introduction

Unlike conventional αβ T cells, γδ T cells express a unique T cell receptor (TCR) composed of γ and δ chains, with minimal or no expression of CD4 or CD8 coreceptors (1, 2). These cells emerge early in thymic development and migrate to tissues in successive waves (3). Human γδ T cells constitute approximately 1 to 5% of circulating T cells, but are enriched in mucous membranes and areas in contact with the external environment (4) where they play a critical role in anti-pathogen control, immunosurveillance and tissue repair (5). Human γδ T cells are classified into distinct subsets based on their TCR Vδ chain usage, with the main subsets being Vγ9Vδ2, Vδ1, and Vδ3 T cells (6).

Among these, Vγ9Vδ2 T cells are the predominant circulating subset, arising early during fetal development and playing a key role in frontline immune defense. Unique to humans and a few other species, such as non-human primates and camelids (7), they detect tumor-derived phosphoantigens via butyrophilin molecules expresses by target cells, particularly BTN3A1 and BTN2A1 (8), which undergo conformational changes upon antigen binding, leading to TCR engagement and cell activation. Beyond TCR stimulation, Vγ9Vδ2 T cell activation is finely tuned by co-stimulatory and co-inhibitory signals, including NK-like receptors such as NKG2D, DNAM-1, and CD16 (9), enabling them to sense stress ligands and antibody-coated targets. This activation triggers robust effector functions, including direct tumor cell lysis through the release of perforin and granzyme, as well as pro-inflammatory cytokine secretion, notably IFN-γ and TNF-α, which further amplify immune responses. Additionally, Vγ9Vδ2 T cells modulate the tumor microenvironment by producing chemokines such as CCL3, CCL4, and CCL5 (10), thereby recruiting and activating other immune players, including αβ T cells (11), NK cells (12) and iNKT cells (13). Their ability to bridge innate and adaptive immunity, combined with their cytotoxic and immunoregulatory properties, positions them as key mediators of both anti-tumor and antimicrobial immunity (14, 15).

Meanwhile, Vδ1 and Vδ3 subsets, primarily located in epithelial and mucosal tissues, contribute to barrier immunity and tissue homeostasis, underscoring the complementary roles of γδ T cell subsets in immune defense (6).

The promising antitumor capabilities of Vγ9Vδ2 T cells have spurred the development of specific immunotherapies targeting this subset. Active immunotherapies, such as administering aminobisphosphonates (e.g., zoledronate or pamidronate) combined with low doses of IL-2, have been shown to induce the activation and proliferation of Vγ9Vδ2 T cells in some cancer patients (16–18). Passive immunotherapies, including adoptive autologous transfer, have demonstrated effective *in vivo* amplification of Vγ9Vδ2 T cells with minimal side effects in renal carcinoma patients (19). Moreover, innovative treatments such as allogenic transfers, which involve donor Vγ9Vδ2 T cells to enhance immune response (20), T cell engagers that redirect T cells to target cancer cells (21) and CAR-Vγ9Vδ2 T cells engineered to recognize specific tumor antigens (22, 23) are showing great promise, with early success in tumor regression and minimal side effects.

However, the therapeutic success of these approaches remains limited, possibly to the exhaustion that Vγ9Vδ2 T cells undergo within the tumor microenvironment (TME). Immune cell exhaustion in the TME results from continuous stimulation by tumor antigens and the presence of immunosuppressive factors like cytokines (e.g., TGF-β and IL-10) and metabolic stress (nutrient deprivation, hypoxia) (24). These factors, along with an increased presence of immunosuppressive cells such as regulatory T cells (Tregs), Tumor-Associated Macrophages (TAMs) and Myeloid-Derived Suppressor Cells (MDSC), disrupt T cell function and promote exhaustion (25). TGF-β and IL-10 contribute by impairing T cell cytotoxicity and promoting the differentiation of naive T cells into Tregs (26). Additionally, these changes lead to metabolic stress, further weakening the antitumor immune response. Immune escape mechanisms triggered by the tumor can suppress T cell effector functions, slow their metabolism, and alter their transcriptomic profiles (27). This exhaustion phenotype was initially characterized in CD8+ T cells during chronic viral infections in mice (28), and later extended to other T cell subtypes, including those involved in bacterial, parasitic, and cancer-related responses in humans (29).

Exhausted T cells have been identified in patient-derived tumor samples and studied *ex vivo*, where their phenotypic markers, functional impairments, metabolic alterations, and transcriptomic profiles have been described (27). Meanwhile, murine and *in vitro* models are increasingly being developed to provide a more comprehensive understanding of the mechanisms underlying T cell exhaustion (30). With growing insights into Vγ9Vδ2 T cell exhaustion, there is a pressing need to develop *in vitro* systems that can replicate this state. Such models would enable high-throughput screening of novel immunotherapies under conditions that closely mimic physiological environments.

In this review, we first summarize the current understanding of Vγ9Vδ2 T cell exhaustion. We then examine various approaches to generate exhausted Vγ9Vδ2 T cells for research purposes, ranging from *ex vivo* cell collection and *in vivo* murine models to cutting-edge *in vitro* experimental setups.

## 2 *Ex vivo* Vγ9Vδ2 T cell exhaustion phenotype

T cell exhaustion was first identified in CD8+ T cells during chronic viral infections in mice (28). It is defined as a dysfunctional state caused by persistent antigenic stimulation, where cells persist but lose their ability to eliminate pathogenic threats (31). Since its initial discovery, exhaustion has been characterized in various animal models and in human chronic viral, bacterial, parasitic infections, and cancers (29). This state is now primarily understood as a loss of effector functions, sustained expression of checkpoint inhibitors (e.g., PD-1, TIM-3, LAG-3, CTLA-4, TIGIT) (27), and more recently, metabolic and epigenetic alterations (30). Although many of these features are observed in exhausted γδ T cells, it remains unclear if the mechanisms of exhaustion differ significantly between CD8+ and γδ T cells. Unraveling the precise mechanisms underlying γδ T cell exhaustion could reveal novel therapeutic opportunities.

Exhausted Vδ2+ T cells have been identified *ex vivo* in a variety of chronic infections and cancers. For example, exposure to *Plasmodium vivax* induces increased expression of exhaustion markers, including PD-1, CTLA-4, TIM-3, and LAG-3, in γδ T cells (32). Similarly, in tuberculosis, Vγ9Vδ2 T cells exhibit elevated PD-1 levels, which are correlated with impaired STAT3 phosphorylation and disrupted IL-2 and IL-23 signaling pathways (33). Preclinical studies have identified exhausted Vγ9Vδ2 T cells in cancer patients undergoing treatment with zoledronate and IL-2, including those with hormone-refractory prostate cancer (34), refractory renal cell carcinoma (35), and breast cancer (36). Single-cell RNA sequencing has also revealed the presence of exhausted Vγ9+ T cells in virus-related cancers, such as head and neck squamous cell carcinoma (HNSCC) and Hodgkin’s lymphoma (HL), with tissue-resident cells displaying higher exhaustion levels than circulating cells (37). In acute myeloid leukemia (AML), exhausted Vδ2+ T cells co-express PD-1 and TIM-3, which is associated with reduced TNF-α and IFN-γ production and increased IL-17 secretion (38, 39). In non-M3 AML (non-acute promyelocytic leukemia), TIGIT+ exhausted Vδ2+ T cells have been linked to poor prognosis (40) while in breast cancer, terminally differentiated Vδ2+ T cells with PD-1 expression are correlated with tumor-draining lymph node invasion (41).

The role of PD-1 in γδ T cell exhaustion remains a subject of debate. For instance, in common variable immunodeficiency (CVID), PD-1hi Vδ2+ T cells have been observed alongside heightened expression of activation markers (e.g., CD38, HLA-DR) on both Vδ2+ and Vδ1+ T cells (42). A higher expression of PD-1 was observed on Vδ2+ cells compared to Vδ1+, with an expansion of Vδ1+ and a loss of Vδ2+. While PD-1 expression is well-considered as a hallmark of CD8+ T cell exhaustion (43–47), recent findings suggest it may not universally signify exhaustion in γδ T cells. Instead, high PD-1 levels in γδ T cells have been associated with retained IFN-γ production, indicating potential roles in activation and differentiation rather than dysfunction (48) but could also be linked to γδ T cell activation and differentiation. Furthermore, PD-1+ Vδ1+ T cells maintain effector responses to TCR signaling and γδ T cells do not respond to PD-1 blockade therapies in certain cancers (49). For instance, in acute dengue infection, impaired IFN-γ production by Vδ2+ T cells has been attributed to TIM-3 expression, rather than PD-1, alongside elevated activation markers (CD38, HLA-DR) (50). *In vitro*, PD-1+ γδ T cells isolated from MMR-deficient colon cancers showed increased reactivity to HLA class I-deficient cancer cell lines compared to those with functional antigen presentation (51). Thus, PD-1 may serve as an activation marker rather than a definitive indicator of γδ T cell exhaustion in specific contexts.

Exhaustion has also been shown to involve distinct subsets. In CD8+ T cells, progressive exhaustion leads to 3 stages ranging from progenitor to terminally exhausted cells (52). Early stages involve metabolic alterations and a loss of IL-2 production, proliferation, and cytotoxicity, while terminally exhausted cells lose TNF-α secretion and, in severe cases, are unable to produce IFN-γ, perforin, or granzyme (52). Progenitor exhausted T cells (Tex) retain self-renewal capabilities and the potential to revert to effector states, unlike terminally exhausted cells (53). Although some markers and transcription factors (e.g., TCF-1) can distinguish Tex subpopulations (54), no consensus has emerged regarding a clear definition of these subsets. This heterogeneity likely reflects the interplay of transcriptional, surface protein, transcriptomic, and epigenetic factors.

Similar heterogeneity has been observed in exhausted γδ T cells. Subsets such as CD160+, CD160+TIGIT+, and CD160+TIGIT+PD-1+ have been identified in HIV+ patients, suggesting that inhibitory receptor profiles can define exhaustion stages (55). Recent *ex vivo* studies in colorectal cancer revealed distinct subpopulations of tumor-infiltrating γδ T cells, including progenitor, intermediate, and terminally exhausted cells, identified via single-cell RNA sequencing and flow cytometry based on PD-1 and TIM-3 expression. These subpopulations were distinguished by differential expression of key exhaustion markers such as PD-1, TIM-3, and LAG-3, as well as markers of activation like CD69 and CD25. Progenitor cells exhibited a less exhausted phenotype with low PD-1 and TIM-3 expression, while intermediate cells displayed a combination of activation and exhaustion markers, and terminally exhausted cells were characterized by high PD-1, TIM-3, and LAG-3 expression, indicating a progressive loss of effector functions (56). However, a comprehensive understanding of these subsets and a standardized classification system remain elusive.

Checkpoint inhibitors and effector dysfunction are not the only hallmarks of exhaustion—transcription factors also play a significant role. For instance, NR4A limits CAR T cell efficacy in solid tumors by promoting exhaustion (57), while BATF suppresses T cell functions in HIV-specific CD8+ T cells (58). Although studies on transcription factors linked to exhaustion in γδ T cells exist, this area is still relatively under-explored compared to the extensive research on αβ T cells. Some studies have suggested that transcription factors like T-bet and Eomes may play a role in γδ T cell exhaustion in chronic conditions such as cancer and infections (59). However, most research on the transcriptomic regulation of exhaustion has relied on murine models, with limited insights derived from *ex vivo* γδ T cells.

## 3 *In vivo* models for studying Vγ9Vδ2 T cell exhaustion

While exhausted T cells were initially identified from human samples of cancer and chronic infections, these methods did not yield sufficient cells for in-depth characterization. To address this limitation, murine models have been developed, providing larger numbers of exhausted cells for detailed analyses of their phenotypic, metabolic, and transcriptomic properties.

Chronic viral infection models in mice have been extensively used to study T cell exhaustion. For example, experiments using adoptive transfer of expanded CD8+ T cells in *Listeria*-infected mice demonstrated that the transcription factor NFAT, when unable to bind AP-1, drives exhaustion-enhancing transcriptional programs (60). Studies using Lymphocytic choriomeningitis virus (LCMV) clone 13 and LCMV-D-infected mice models, with persistent infection, have revealed key insights into exhaustion mechanisms (61) (28) (62–65). These studies showed that IRF4 promotes CD8+ T cell exhaustion by limiting memory cell development (62), while T-bet represses PD-1 expression and supports effector functions during chronic infection (63). Such experiments often involve analyzing antigen-specific T cells from spleens, lymph nodes, or tissues at various stages of infection Murine models remain the most commonly used approach for studying T cell exhaustion due to their flexibility, but translating findings from mice to humans presents challenges, particularly for γδ T cells (66).

Unlike CD8+ and CD4+ T cells, γδ T cells exhibit substantial differences between humans and mice. Human γδ T cells differ in their γ and δ TCR chain recombination, as well as the stoichiometry of the CD3 complex associated with the TCR (67, 68). Additionally, the human Vγ9Vδ2 T cell subset, which is uniquely capable of detecting phosphoantigens via butyrophilin molecules, has no counterpart in mice. These differences complicate the use of murine models for studying Vγ9Vδ2 T cell exhaustion. To date, the only *in vivo* study describing exhausted Vγ9Vδ2 T cells utilized a non-human primate model (Cynomolgus monkey) that received repeated injections of the synthetic phosphoantigen 3-(bromomethyl)-3-butanol-1-yl-diphosphate (BrHPP) (69). This study demonstrated that repeated infusions of BrHPP and IL-2 are increasingly less efficient for inducing peripheral Vγ9Vδ2 T cell expansion, a phenomenon referred to as tachyphylaxis, suggesting a gradual exhaustion upon repeated injections.

To overcome these challenges, there is a growing need to develop humanized mouse models that can sustain the presence of human Vγ9Vδ2 T cells for sufficient durations to study their exhaustion features. Humanized mouse models—immunodeficient mice engrafted with human tumors and immune cells—are increasingly used in immuno-oncology research due to their translational potential (70). However, these models face limitations, including the lack of HLA molecules, restricted development of mature innate immune cells, and limited capacity to generate antigen-specific antibody responses. Efforts are underway to create advanced humanized models that more accurately mimic human innate and adaptive immunity while supporting the long-term survival of human γδ T cells.

Similar to NK cells, γδ T cell survival and proliferation in *in vivo* experiments might be dependent on cytokines supplementation (IL-2, IL-15, IL-21) (71, 72). For instance, injecting IL-15-IL-15Rα/Fc complexes into CD34+ hematopoietic stem and progenitor cell (HSPC)-engrafted BRG mice promotes the transient development of functional human NK cells (73) and may also support γδ T cells. Transgenic expression of IL-15 has enabled functional NK cell development in BRGS (74), CD34^+^ HSPC-engrafted NSG (75) or NOG mice (76), which could similarly benefit Vγ9Vδ2 T cell studies. These models could potentially facilitate the generation of functional Vγ9Vδ2 T cells *in vivo* for further research.

The humanized bone marrow-liver-thymus (BLT) mouse model contains a nearly fully functional human immune system. The model provided insights into the dynamics of γδ T cell responses during HIV infection, highlighting their role in both immune surveillance and potential exhaustion in the context of chronic infection. Indeed, researchers found that HIV infection in BLT humanized mice impaired the *ex vivo* expansion of Vδ2 T cells, like in HIV-positive individuals (77). This model could be adapted to induce exhaustion in Vγ9Vδ2 T cells *in vivo*.

However, the complexity and time-consuming nature of engrafting human immune components into immunodeficient mice (70) make these models less ideal for routine study of Vγ9Vδ2 T cell exhaustion. Consequently, *in vitro* models are crucial and should be developed alongside advancements in humanized mouse systems. These complementary approaches can collectively enhance our understanding of Vγ9Vδ2 T cell exhaustion and accelerate the evaluation of therapeutic strategies.

## 4 Novel *in vitro* models for generating exhausted Vγ9Vδ2 T cells

Advancements in *ex vivo* expansion techniques have made it easier to generate large numbers of Vγ9Vδ2 T cells from human peripheral blood mononuclear cells (PBMCs). Using natural phosphoantigens like HMBPP, synthetic alternatives such as BrHPP, or aminobisphosphonates like zoledronate, researchers can produce sufficient cell quantities for *in vitro* studies of immunotherapies targeting these cells (78–80). *In vitro* models of exhausted T cells (Tex) are highly customizable and allow for the rapid generation of large cellular yields, making them ideal for high-throughput experiments. These models aim to create conditions that mimic the exhaustion phenotype observed in *ex vivo* cells as closely as possible by incorporating specific environmental and stimulatory factors into the culture medium. However, the methods used for expansion may introduce bias in the overall condition of the cells.

Various protocols have been developed to induce exhaustion in CD8+ and CD4+ T cells. The most common method involves persistent stimulation with anti-CD3/CD28 beads or bead-coated plates, which has been shown to increase exhaustion marker expression (e.g., PD-1, LAG-3, TIM-3), reduce cytokine production, and impair cytotoxic capabilities (81). Subsequent studies have analyzed the transcriptomic and metabolic profiles of these cells and compared them to *in vivo* exhausted T cells (82). However, these comparisons revealed discrepancies, suggesting that *in vitro* models do not entirely replicate the characteristics of *in vivo* Tex cells (66).

To address these limitations, recent refinements in exhaustion protocols have included a two-phase stimulation process: two days of anti-CD3/CD28 bead stimulation with IL-2, followed by eight days of anti-CD3 antibody-coated beads with IL-2 (81). Other approaches have incorporated hypoxic conditions (1.5% oxygen) during stimulation to better mimic the tumor microenvironment (83). Both strategies have resulted in cells that exhibit a more pronounced exhaustion phenotype, with transcriptional profiles more closely resembling those of terminally exhausted T cells observed in a B16 melanoma *in vivo* model (84).

Despite these advances, these models have primarily been developed for conventional T cells, and few have been optimized for Vγ9Vδ2 T cells. Notably, Vγ9Vδ2 T cells appear to have a higher sensitivity to persistent stimulation *in vitro* and may not tolerate existing protocols designed for αβ T cells. This highlights the need for tailored methods specific to Vγ9Vδ2 T cells, such as phosphoantigen-induced persistent stimulation, which mirrors conditions observed in patients treated with zoledronate and low-dose IL-2 (17, 18).

The absence of murine models and detailed transcriptomic characterization of *in vivo* exhausted Vγ9Vδ2 T cells further complicates efforts to accurately replicate their “real” exhaustion phenotype *in vitro*. Developing specific protocols that account for these unique challenges is essential for advancing the study of Vγ9Vδ2 T cell exhaustion and evaluating new immunotherapies.

## 5 Discussion

Each model used to investigate Vγ9Vδ2 T cell exhaustion exhibits distinct characteristics and presents both benefits and drawbacks that are summarized in **Table 1**. These models provide valuable insights into the mechanisms underlying exhaustion, each with its own set of strengths and limitations, which should be carefully considered when planning future research.

**Table 1:** Summary of the different types of exhaustion models, along with their key characteristics, advantages and limitations.

Model Type	Key Characteristics	Advantages	Limitations	References
*Ex vivo* Models	- Exhausted cells collected directly from patient samples - Provide insights into actual tumor microenvironment	- Reflect “real” exhaustion characteristics - Closely mimic physiological conditions - Allow direct study of human cells	- Limited availability of patient samples - Limited variability in the types and grades of accessible tumors - Low yield of exhausted cells - Heterogeneous cell population	(34–36)
Murine Models	- Use of chronic viral or cancer models to induce T cell exhaustion in mice - Allow exploration of mechanisms and therapies	- Flexible and well-established systems - Facilitate transcriptomic and metabolic studies - No reliance on human samples	- Vγ9Vδ2 T cells are absent in mice - Differences in TCR recombination and immune responses compared to humans - Absence of human tumor microenvironment	(60–64)
Non-Human Primate Models	- Use of primates to induce exhaustion (e.g., repeated phosphoantigen injections) - Closest *in vivo* approximation of human immune responses	- Closely resemble human immune system - Enable *in vivo* study of Vγ9Vδ2 T cell exhaustion	- Expensive and complex to implement - Few robust and relevant tumor models in primates - Ethical considerations - Not scalable for routine studies	(69)
Humanized Mouse Models	- Immunodeficient mice engrafted with human tumors and immune cells - Allow partial reconstitution of human immune systems	- Enable study of human Vγ9Vδ2 T cells - Potential for clinical translation - Can support sustained immune responses with IL-15	- Limited cell survival without cytokine supplementation - Incomplete representation of human immunity - Expensive and labor-intensive	(73–77)
*In vitro* Models	- Exhaustion induced in T cells expanded from PBMCs using sustained stimulation, hypoxia, or nutrient deprivation	- High yield of cells (up to 10^8^) - Highly customizable - Suitable for high-throughput screening of therapies	- May not fully replicate tumor microenvironment - Lack of interactions with other immune subtypes - Limited data for Vγ9Vδ2-specific exhaustion protocols	(81–83)

As highlighted in this review, current knowledge on Vγ9Vδ2 T cell exhaustion primarily comes from studies on chronic infections, cancers, and immunotherapy trials, with *ex vivo* analyses providing valuable insights into their behavior in the tumor microenvironment. However, limitations in tissue availability and cell numbers hinder downstream applications and the development of *in vivo* and *in vitro* models. Murine models, while useful for studying general T cell exhaustion, lack Vγ9Vδ2 T cells, and non-human primates, though suitable, are costly and complex. Humanized mice offer a partial solution but may require further optimization such as cytokine supplementation in order to improve γδ T cell survival, which could introduce biases. *In vitro* models show promise for rapid and scalable research, enabling detailed characterization and testing of therapies, but they fail to capture the full complexity of the tumor microenvironment and require further refinement to minimize biases arising from culture conditions. Developing tailored *in vitro* protocols and leveraging complementary *in vivo* models are critical for advancing the understanding of Vγ9Vδ2 T cell exhaustion and optimizing immunotherapies.

To extensively utilize *in vitro* models in the early stages of developing novel immunotherapies, it is essential to create models that replicate the exact characteristics and exhaustion profiles observed in patient-derived cells. However, this poses significant challenges. Each type of cancer induces unique phenotypic and transcriptomic profiles in exhausted cells, and there is substantial variability in the stages of exhaustion, each defined by distinct phenotypes and a high degree of heterogeneity within the exhausted cell population. These complexities make it nearly impossible to generate models that perfectly mimic physiological conditions.

One potential solution is to develop a comprehensive catalog of exhaustion models, standardized and characterized to reflect the different exhaustion profiles observed across various cancers and cell types. At present, the focus should be on refining *in vitro* models to generate cells that exhibit most or all of the phenotypic and transcriptomic features of *in vivo* exhaustion. Such models could significantly accelerate research on innovative immunotherapies by enabling early-stage testing in conditions that closely resemble the tumor microenvironment.

For therapies targeting Vγ9Vδ2 T cells, it is imperative to design robust and consistent *in vitro* models specifically tailored to this subpopulation. Additionally, advancements in humanized mouse models are necessary to sustain Vγ9Vδ2 T cells and enable further characterization of their exhaustion properties. These models would provide valuable comparative data to inform the development of more precise *in vitro* protocols.

We firmly believe that *in vitro* exhaustion models hold the greatest potential for driving the early-stage development of novel immunotherapies. By combining these models with complementary *in vivo* systems, researchers can build a comprehensive framework for testing and optimizing therapeutic strategies.

In conclusion, a thorough understanding of Vγ9Vδ2 T cell exhaustion is essential for the development of effective immunotherapies. However, current models encounter significant challenges, such as limitations in tissue availability and model accuracy. *In vivo* models are critical for studying immune-tumor interactions and can provide valuable insights into the complexity of the tumor microenvironment. However, they may have limitations when it comes to studying human-specific immune populations, such as Vγ9Vδ2 T cells, which are absent in mice. This represents a challenge to fully understand the contribution of these immune cell effectors in cancer and immune responses in human patients. In contrast, *in vitro* models provide more controlled environments that enable detailed studies of specific cellular interactions, though they do not replicate the full complexity of the tumor microenvironment nor the systemic interactions which are observed *in vivo*.

To overcome this, refining *in vitro* protocols specific to Vγ9Vδ2 T cells is critical for a deeper understanding of exhaustion dynamics. By standardizing and optimizing these models, particularly those focused on Vγ9Vδ2 T cells, researchers can accelerate the development of innovative therapies. Ultimately, integrating these models will create a robust framework to advance immunotherapy strategies and enhance patient outcomes.
